# Supplementary material for: Effects of Community-Led Total Sanitation (CLTS) Boosting and Household Factors on Latrine Ownership in Siaya County, Kenya
Source: Int J Environ Res Public Health. 2023 Sep 18;20(18):6781. doi: 10.3390/ijerph20186781 (PMC10531019; doi:10.3390/ijerph20186781)
Supplement: Supplementary file 1 [file ijerph-20-06781-s001.zip › Supplement 1. Baseline Questionnaire.pdf]

HOUSEHOLD QUESTIONNAIRE & REGISTRATION

GENERAL INFORMATION

CONSENT

ENSURE CONSENT IS OBTAINED AND SIGNED

Consent

Yes = 1

No = 0

Questionnaire Code: _ _ _ _ _

Date of interview (dd/mm/yyyy) ____/_____/_____

Time of interview ___________

Household Code: ________________________

Ask to speak with the Head of the Household. If they are unavailable, ask to speak with: 1-female head of household, 2-adult child, 3-grandparent.

Sub County: __________________________________

Village cluster and Name: _______________________

HOUSEHOLD BACKGROUND INFORMATION

1. How many households are in this compound? ________ Households
2. How many people live in this compound? ________ People
3. What village do you live in? _______________________________
4. How many primary school age children between the ages of 5 and 18 years live in this household? ________ children

Please fill in table for above children

Age (years)	Sex M=1 F=2	Does s/he attend school? (Y/N)	Has s/he had any of the following in the past week? **O?** (read options) 1-Diarrhea 2-Intest. Worms 3-Typhoid 4-Amoeba 5-Others_______	Has s/he missed school in the past two weeks? (Y/N/DK)	Why has s/he missed school in the past two weeks? 1 – illness 2 – work 3 – taking care of sibling 4 – caring for sick family member	If illness, what type of illness? 1 – Diarrhea 2 – Stomachache 3 – Malaria 4 – Cough 5 – Typhoid 6 – vomiting 7 - Headache 8- Other_____

					5 – other______	

5. How many children under 5 years of age live in this household? *Record number in household*

_______ number of children

Please fill in below table for above children

Age (months)	Sex (M/F)	Has s/he had any of the following in the past week? (read responses)<br>1-Diarrhea<br>2-Cough<br>3-Feaver<br>4-Vomiting	Has s/he had diarrhea in the past week??<br>1) Yes<br>2) No<br>3) DK	Was there blood in the stool?<br>1) Yes<br>2) No<br>3) DK	Did s/he visit the clinic in the past two weeks?<br>1) Yes<br>2) No<br>3) DK	Did s/he visit the clinic because of diarrhea in the past two weeks?<br>1) Yes<br>2) No<br>99) DK

6. How many people live in this household? *(The definition of a household is people eating from the same kitchen/pot) Record number in household*

_____ Number of people

7. Respondent sex ***(DON'T ASK, CIRCLE CORRECT RESPONSE)***

1) Male
2) Female

How old are you? ________

Complete the following table:

	Age? *(record age or status)*<br>=deceased<br>=N/A	Marital Status<br>1=Married<br>2=Single<br>3=Separated<br>4=Widowed<br>5=Divorced<br>6=N/A	Highest level of education<br>0=no education<br>1=some primary<br>2=finished primary<br>3=some secondary<br>4=finished secondary<br>5=some tertiary	Able to read?<br>1) yes<br>2) no	Has s/he had diarrhea in the past week?<br>1) yes<br>2) no<br>3) DK

			6=completed cert., diploma, higher diploma, etc		
Male head of HH					
Female head of HH					

SANITATION INFORMATION

Now I am going to ask you a few questions about sanitation and personal hygiene.

8. Do you have a toilet facility in this compound?

1) Yes ‣ skip to Q11
2) No

9. Why is there no toilet in this compound? *(Multiple responses possible)*

1) Can't afford it
2) Soil too loose / rocky
3) Do not need one
4) Collapsed
5) Full
6) Prefer outdoors
6) Other, specify ________________________________

10. Where do you go to make a long call (defecate) if you don't have a latrine at home?

1) On compound grounds somewhere
2) Behind the latrine
3) Friend's house
4) Public latrine
5) Neighbor's house
6) Bush/field
7) Other, specify: __________________

11. If you have visitors, where do they go for a long call (defecate) if you don't have a latrine at home?

1) On compound grounds somewhere
2) Behind the latrine
3) Friend's house
4) Public latrine
5) Neighbor's house
6) Bush/field
7) Other, specify

12. How many latrines on this compound are currently being used?_____ functioning latrines

If more than one facility is functioning in the compound note them down and who uses them

13. Do you share this/these toilet(s) with other households?

1) Yes
2) No

14. How many other households use this/these toilet(s)?________ households

15. When did you build this latrine (Ask only if latrine is observed)?

______months ago *(conversion to years)*

______ years ago

______Don't know

16. When was the last time this/these toilet(s) was/were improved or constructed?

______ months ago *(conversion to years)*

______ years ago

______Don't know

17. Is there anyone in this household, including children, who does not regularly use the latrine?

1) Yes
2) No

18. Who doesn't regularly use the latrine? (*multiple responses possible)*

Children <2 years

Children <5 years

Children 5 – 15 years

Female adults

Male adults

No one uses latrine

Other ___________________

19. Why do these people not use the latrine?

Children too small

No latrine at home

Not well kept

Distance from the compound

Fear, dislike

Other________________

20. How do you dispose of the feces of your child/children under 5 years?
   1) Leave it in the yard / do nothing
   2) Put in the latrine
   3) Bury it
   5) Other, specify _____________________
   6) Don't know

21. Have you ever heard of community led total sanitation (CLTS)?
   1) yes
   2) No

   If yes, can you describe for me what it is

   ____

22. Have you attended any of CLTS training sessions?
   1) Yes
   2) No ‣ skip to Q 42

23. Who facilitated the CLTS training session in this village?
   1) Government officers (public health officers)
   2) None Governmental Organization
   3) Other,_______________________________

24. In your opinion, do you think CLTS is helping your village to have latrines?
   1) Yes
   2) No

25. Do you think CLTS is a good intervention to help people build latrines in your village?

1) Yes
2) No

Why do you say so? ____

26. In your opinion, what worked well during the CLTS triggering sessions?

Transect walk/mapping of open defecation areas
Shit calculation
Medical expenses calculation
Demonstration on disease pathways
Other, Specify ______________________________________

27. In your opinion, what did not work well during the CLTS triggering sessions?

Transect walk/mapping of open defecation areas
Shit calculation
Medical expenses calculation
Demonstration on disease pathways
Other, Specify ______________________________________

28. What did you like most about the CLTS process?

Transect walk/mapping of open defecation areas
Shit calculation
Medical expenses calculation
Demonstration on disease pathways
Other, Specify ______________________________________

29. What did you not like most about the CLTS process?

Transect walk/mapping of open defecation areas
Shit calculation
Medical expenses calculation
Demonstration on disease pathways
Other, Specify ______________________________________

30. Did you ever built/construct a latrine after the CLTS training?

1) Yes
2) No

31. What made you built/construct a latrine? __________________________________

32. Are you satisfied with the type of latrine you have constructed?

1. Very satisfied 2. Satisfied 3. Not satisfied 4. Don't Know

33. Which type of latrine would you prefer?
   1) VIP latrine
   2) Water closet
   3) Ecosan
   4) Sanplat
   5) Other, describe ______________________________________

34. Where you ever told about the types of latrines to construct?
   1) Yes
   2) No
35. Did you have a latrine prior to the CLTS training/triggering?
   1) Yes
   2) No
36. Were you ever told about punishment to those who do not have or build latrine?
   1) Yes
   2) No
37. What are the forms of punishment practiced in this village to those who do not have latrines? ....
…………………………………………………………………………………………….
38. Does the punishment make them build latrines?
39. Has there been follow ups after the CLTS training
   1) Yes
   2) No
40. How frequent are the follow-ups
   1) Very frequent
   2) Once a month
   3) Not frequent
   4) Don’t Know
   5) Never
41. How are you currently using the information obtained during CLTS training?
42. Do you think the construction of latrines helps in prevention of diseases?
   1) Yes
   2) No
43. Can you name for me some of these diseases?
   1) Diarrhea
   2) Typhoid

3) Cholera
4) Intestinal worms
5) Other, Specify___________________________

44. Why do you think people practice open defecation in this village?

HOUSEHOLD / DWELLING INFORMATION

Now I am going to ask you a few questions about your household and dwelling.

45. What type of fuel does your household **mainly** use for cooking? *(Choose one)*

Electricity

Natural Gas

Biogas

Paraffin / Kerosene

Charcoal

Firewood / straw

Dung

Other__________

46. What is the primary method for lighting the household dwelling(s)? *(This is lighting in the main room, NOT in the kitchen. Choose one)*

1) Paraffin (tin and wick)
2) Paraffin (Hurricane lantern)
3) Electricity
4) Solar lamp
5) Pressure lamp
6) Gas
7) Other, specify ________________________

47. How many of the following does the household own? *(Write the number owned next to each asset. Read SENSITIVELY)*

Type of animal	*Number owned*
Poultry	
Cattle	
Goats	
Sheep	
Pigs	
Donkeys	
Other, Specify	

48. Which of the following items does the household have in working order?

Mobile/telephone	Yes No
Television	Yes No
Sewing Machine	Yes No
Posho Mill	Yes No
Ox-Plough	Yes No
Gas/Electric cooker	Yes No
Bicycle	Yes No
Radio	Yes No
Motorcycle	Yes No
Motor vehicle	Yes No
Boat	Yes No
Other, specify	

OBSERVATION

Main roofing wall and floor material for the household's dwelling (*if more than one building in compound, list the structure that the interviewee lives in)*:

Roof	*Wall*	*Floor*
1) Grass thatch	1) Mud	1) Dung / Earthen
2) Cement	2) Cement	2) Cement / plaster
3) Tiles	3) Bricks / Blocks	3) Tile
4) Timber	4) Timber	4) Timber
5) Iron sheet	5) Metal/iron sheet	
6) Asbestos sheets		

State of repair of the dwelling

1) Completely dilapidated/ not livable
2) Needs major repairs
3) Needs no repairs / minor repairs
4) Being repaired now
5) New home/ under construction

Observed feces around compound. 1) Yes 2) No

Can you please show me the types of latrines that you are using?

Number of functioning latrines observed around compound ______ "doors"

Type of toilet facility in compound. If more than one, determine newest facility.

Flush Toilet
Traditional Pit Latrine
Traditional Pit with EcoSan
Ventilated Improved Pit Latrine
Ventilated Improved Pit with Ecosan
No Facility / Bush / Field
Above ground vault
Slab only
Other Specify____

Condition of the latrine most frequently used by members of the household

Smell	1) No smell	2) smell inside	3) smell outside
Cleanliness	1) Clean	2) Slightly dirty	3) Feces/Very dirty
Flies	1) No flies	2) A few flies	3) Many flies
Superstructure	1) No cracks	2) Cracks	3) Visible holes

Condition of slab 1) No cracks 2) Some cracks 3) Pit visible

Door 1) Door closes completely 2) Door closes, but not completely 3) No door

Does the latrine hole have a separate lid? 1) Yes 2) No

Was the lid covering the hole at the time of the site visit? 1) Yes 2) No

Is the pit partially full or almost full?

1) Pit is empty or nearly empty
2) Pit is partially full w/ feces
3) Feces are visible at or near the top of the pit
4) Not applicable

Describe the materials used to construct the latrines ________________________________

Thank you for your time. Do you have any questions?
