# Supplementary material for: Effects of Community-Led Total Sanitation (CLTS) Boosting and Household Factors on Latrine Ownership in Siaya County, Kenya
Source: Int J Environ Res Public Health. 2023 Sep 18;20(18):6781. doi: 10.3390/ijerph20186781 (PMC10531019; doi:10.3390/ijerph20186781)
Supplement: Supplementary file 1 [file ijerph-20-06781-s001.zip › Supplement 2. Follow-up Questionnaire.pdf]

HOUSEHOLD QUESTIONNAIRE

GENERAL INFORMATION

The purpose of this data collection is to follow up on a previous household registration/baseline data collection. The baseline information collected includes those who have and those who do not have latrines. For this follow up exercise, we will only collect information from those who attended CLTS triggering sessions/trainings in the community.

CONSENT

ENSURE CONSENT IS OBTAINED AND SIGNED

Consent

Yes = 1

No = 0

Questionnaire Code: _ _ _ _ _

Date of interview (dd/mm/yyyy) ____/_____/_____

Time of interview ___________

Compound/Household Code: ________________________

Sub County: _________________________________

Village cluster and Name: _______________________

Ask to speak with the Head of the Household.

HOUSEHOLD BACKGROUND INFORMATION

1. How many households are in this compound? ________ Households
2. How many people live in this compound? ________ People
3. How many children under 5 years of age live in this household? *Record number in household*

_______ number of children

Please fill in below table for above children

Age (months)	Sex (M/F)	Has s/he had any of the following in the past week? (read responses)<br>1-Diarrhea<br>2-Cough<br>3-Fever<br>4-Vomiting	Has s/he had diarrhea in the past week?**?**<br>1) Yes<br>2) No<br>9) DK	Was there blood in the stool?<br>1) Yes<br>2) No<br>9) DK	Did s/he visit the clinic in the past two weeks?<br>1) Yes<br>2) No<br>9) DK	Did s/he visit the clinic because of diarrhea in the past two weeks?<br>1) Yes<br>2) No<br>9) DK

4. How many people live in this household? *(The definition of a household is people eating from the same kitchen/pot) Record number in household*

_____ Number of people

5. Respondent sex ***(DON'T ASK, CIRCLE CORRECT RESPONSE) (Please ensure you speak to the household head)***

1) Male
2) Female

How old are you? ________

Complete the following table:

	Age? *(record age or status)*<br>=deceased<br>=N/A	Marital Status<br>1=Married<br>2=Single<br>3=Separated<br>4=Widowed<br>5=Divorced<br>6=N/A	Highest level of education<br>0=no education<br>1=some primary<br>2=finished primary<br>3=some secondary<br>4=finished secondary<br>5=some tertiary<br>6=completed cert., diploma, higher diploma, etc	Able to read?<br>1) yes<br>2) no	Has s/he had diarrhea in the past week?<br>1) yes<br>2) no<br>3) DK
Male head of HH					
Female head of HH					
Others (specify e.g child headed etc)					

CLTS/SANITATION INFORMATION

Now I am going to ask you a few questions about sanitation and personal hygiene.

6. Have you ever heard of community led total sanitation (CLTS)?
   1) yes
   2) No

   If yes, can you describe for me what it is ___________________________________
7. Have you or any member of your family attended any of CLTS training sessions?
   1) Yes
   2) No

   If yes, when was this? …………….. (state the month and year)
8. Who attended the CLTS training/triggering session?
   1) My husband
   2) My wife
   3) My child (How old was the child_______ years)
   4) Nobody
   5) Others (specify)_____________________________

9. Who facilitated/implemented the CLTS training/triggering session in this village?

1) Government officers (public health officers)
2) Nongovernmental Organization
3) Other, (specify) ___________________________

10. Which language did the facilitators use during the CLTS training/triggering session?

1) Dholuo
2) Kiswahili
3) English
4) Other, specify. ____________________________________

11. Did you ever build a latrine after the CLTS training/triggering session?

1) Yes
2) No ⟶ skip to Q 16

If yes, after how long? ______________ Days (after triggering)

12. What made you to build the latrine?

____

____

____

13. Is the latrine still present? (Observe presence of latrine)

1) Yes ⟶ skip to Q 15
2) No

If No, what happened to the latrine you built? _____________________________

____

14. Why have you not built another latrine? __________________________________

____

____

15. Is the latrine being used by all the members of the family?

1) Yes
2) No

If No, who is not using and why?

____

16. Do you think CLTS is a good intervention to help people build latrines in your village?

1) Yes
2) No

Why do you say so? ____

17. In your opinion, what worked well during the CLTS triggering sessions?

1) Transect walk/mapping of open defecation areas
2) Shit calculation
3) Medical expenses calculation
4) Demonstration on disease pathways
5) Other, Specify ______________________________________

Why do you say so?

____

____

18. In your opinion, what did not work well during the CLTS triggering sessions?

1) Transect walk/mapping of open defecation areas
2) Shit calculation
3) Medical expenses calculation
4) Demonstration on disease pathways
5) Other, Specify ______________________________________

Why do you say so? ____

19. Are you satisfied with the type of latrine you have constructed?

1. Very satisfied 2. Satisfied 3. Not satisfied 4. Don't Know

20. Which type of latrine would you prefer?

1) VIP latrine
2) Water closet
3) Ecosan
4) Sanplat
5) Other, describe ____

21. Did you have a latrine prior to the CLTS training/triggering session?
    1) Yes
    2) No
22. Were you ever told about the types of latrines to construct?
    1) Yes
    2) No
23. Were you ever told about punishment to those who do not have or build latrine?
    1) Yes
    2) No
24. What are the forms of punishment were you told?

25. What forms of punishment are being practiced in this village to those who do not have
latrines? ....
…………………………………………………………………………………………
26. Were you ever punished for not building a latrine?
    1) Yes
    2) No

    If yes, what type of punishment was it? ……………………………………
27. Did the fear of punishment make you build latrine?
    1) Yes
    2) No
28. Has there been follow ups after the CLTS trainings/triggering
    1) Yes
    2) No
29. How frequent are the follow-ups
    1) Once a weekly
    2) Twice a week
    3) Once a month
    4) Twice monthly
    5) Once a year
    6) Don't Know
    7) Never

30. Who has been conducting the CLTS follow ups? (*multiple response possible*)
    1) Government officers
    2) NGO staff
    3) Nobody
    4) Community members/natural leaders
    5) CHVs/CHWs
    6) Other, specify. ____

31. Do you think the construction of latrines helps in prevention of diseases?
    1) Yes
    2) No

32. Can you name for me some of these diseases?
    1) Diarrhea
    2) Typhoid
    3) Cholera
    4) Intestinal worms
    5) Other, Specify______________________________

33. Why do you think people practice open defecation in this village? _______________

____

____

34. What are the situations that have made you or other people not to use latrines in this village?
    1) Lack of money to construct latrines/poverty
    2) Lack of information of type of latrines to construct
    3) Many bushes/open fields
    4) Latrine filled up
    5) Soil loose/rocky
    6) Other, specify. ____

HOUSEHOLD / DWELLING INFORMATION

Now I am going to ask you a few questions about your household and dwelling.

35. What type of fuel does your household **mainly** use for cooking? *(Choose one)*
    1) Electricity
    2) Natural Gas
    3) Biogas
    4) Paraffin / Kerosene
    5) Charcoal
    6) Firewood / straw
    7) Dung
    8) Other__________

36. What is the primary method for lighting the household dwelling(s)? *(This is lighting in the main room, NOT in the kitchen. Choose one)*
    1) Paraffin (tin and wick)
    2) Paraffin (Hurricane lantern)
    3) Electricity
    4) Solar lamp
    5) Pressure lamp
    6) Gas
    7) Other, specify ________________________

37. How many of the following does the household own? *(Write the number owned next to each asset. Read SENSITIVELY)*

Type of animal	*Number owned*
Poultry	
Cattle	
Goats	
Sheep	
Pigs	
Donkeys	
Other, Specify	

38. Which of the following items does the household have in working order?

Mobile/telephone	Yes No

Television	Yes No
Posho Mill	Yes No
Gas/Electric cooker	Yes No
Motorcycle	Yes No
Motor vehicle	Yes No
Other, specify	

OBSERVATION

1. Main roofing wall and floor material for the latrine:

Roof	*Wall*	*Floor*
1) Grass thatch	1) Mud	1) Dung / Earthen
2) Cement	2) Cement	2) Cement /plaster
3) Tiles	3) Bricks / Blocks	3) Tile
4) Timber	4) Timber	4) Timber
5) Iron sheet	5) Metal/iron sheet	
6) No roof	6) Polythene paper	
6) Polythene paper	7) Bed nets/sheets	
7) Bed nets/sheet	8) Gunny bags	

Others, specify: ____-

2. State of repair of the latrine
   1) Completely dilapidated/ not livable
   2) Needs major repairs
   3) Needs no repairs / minor repairs
   4) Being repaired now
   5) New latrine under construction

3. Observed feces around compound. 1) Yes 2) No
4. Can you please show me the types of latrines that you are using?
5. Number of functioning latrines observed around compound ______ "doors"
6. Type of toilet facility in compound. If more than one, determine newest facility.
   1) Flush Toilet
   2) Traditional Pit Latrine
   3) Traditional Pit with EcoSan
   4) Ventilated Improved Pit Latrine

5) Ventilated Improved Pit with Ecosan
6) No Facility / Bush / Field
7) Above ground vault
8) Slab only

Other Specify____

7. Condition of the latrine most frequently used by members of the household

Smell	1) No smell	2) smell inside	3) smell outside
Cleanliness	1) Clean	2) Slightly dirty	3) Feces/Very dirty
Flies	1) No flies	2) A few flies	3) Many flies
Superstructure	1) No cracks	2) Cracks	3) Visible holes

8. Condition of slab 1) No cracks 2) Some cracks 3) Pit visible

9. Condition of Door 1) Door closes completely 2) Door closes, but not completely 3) No door

10. Does the latrine hole have a separate lid? 1) Yes 2) No

11. Was the lid covering the hole at the time of the site visit? 1) Yes 2) No

12. What is the condition of the pit?
1) The pit is full
2) Pit is empty or nearly empty
2) Pit is partially full w/ feces
3) Feces are visible at or near the top of the pit
4) Not applicable

Thank you for your time. Do you have any questions?
