# Supplementary material for: Effects of Community-Led Total Sanitation (CLTS) Boosting and Household Factors on Latrine Ownership in Siaya County, Kenya
Source: Int J Environ Res Public Health. 2023 Sep 18;20(18):6781. doi: 10.3390/ijerph20186781 (PMC10531019; doi:10.3390/ijerph20186781)
Supplement: Supplementary file 1 [file ijerph-20-06781-s001.zip › Supplement 3. example_IC_used.pdf]

NAGASAKI UNIVERSITY
Institute of Tropical Medicine
Department of Eco-epidemiology

Informed Consent form for Households

Name of Principal Investigator: Job Wasonga
Name of Organization: Nagasaki University
Name of Sponsor: Japanese Society for Promotion of Science
Name of Proposal and version: Sustainability Challenges to implementation of CLTS in Siaya County Version 2.0

This Informed Consent Form has two parts:

- **Information Sheet (to share information about the research with you)**
- **Certificate of Consent (for signatures or verbal acceptance if you agree to take part)**

You will be given a copy of the full Informed Consent Form

PART I: Information Sheet

Introduction

I am [redacted], collecting data for a student of Nagasaki University. We are doing research a longitudinal study on sustainability challenges in implementation of community- led total sanitation in this County (Siaya County). I am going to give you information and invite you to be part of this research. You do not have to decide today whether or not you will participate in the research. Before you decide, you can talk to anyone you feel comfortable with about the research.

There may be some words that you do not understand. Please ask me to stop as we go through the information and I will take time to explain. If you have questions later, you can ask me or the main researcher whom contacts I shall give you.

Purpose of the research

Sanitation is one of the major ways of controlling diarrhea. Sanitation involves construction and proper use of latrines in our households or homestead. Unfortunately, may people do not use latrines while answering a call of nature. Because of this none usage of latrines by majority of people, an approach known as CLTS was developed in Bangladesh and adopted by Kenya Government to improve on latrine access and usage. CLTS is a participatory approach and is based on the principle that communities must be empowered to stop open defecation (OD) and to build and use latrines without the support of any external help. The approach uses 'shaming and disgust' to make people construct latrines. Unfortunately, many people revert to open defecation after sometime despite having been exposed to CLTS approach. It is this reason why we are doing this research to find out sustainability challenges that face the implementation of CLTS approach and make people go back to not using latrines while attending call of nature.

Type of Research Intervention

This research will involve household interview and registration as well as follow-up visits to the same households over a period of time

Participant selection

We are interviewing and registering households in this village that have been randomly selected to participate in this research. Not all households have been selected to participate *but just a few of which* your house is one of them.

Voluntary Participation

Your participation in this research is entirely voluntary. It is your choice whether to participate or not. Whether you choose to participate or not, we will not force you to take part in this and you may change your mind later and stop participating even if you agreed earlier.

Duration

The research takes place over 12 months in total. During this time, the research assistants may visit your household after every 4 months to collect data after which the research will end.

Risks

Although there may be no risks involved in terms of bodily harm by participating in this study, some questions may be to probing and touching on your personal life which may cause some discomfort

Benefits

There may not be any benefit for you but your participation is likely to help us find the answer to the research question and solve the problems that your community has been facing concerning sustainability of the sanitation access.

Reimbursements

You will not be given any money or gifts or any other thing as inducement to take part in this research.

Confidentiality

The information that we collect from this research project will be kept confidential. Information about you that will be collected during the research will be put away and no-one but the researchers will be able to see it. Any information about you will have a number on it instead of your name. Only the researchers will know what your number is and we will lock that information up with a lock and key. It will not be shared with or given to anyone except co- investigators, Prof. Mohamed Karama and Prof. Satoshi Kaneko

Did you understand the procedures that we will be using to make sure that any information that we as researchers collect about you will remain confidential? Do you have any questions about them?

Sharing the Results

The knowledge that we get from doing this research will be shared with you through community meetings before it is made widely available to the public. Confidential information will not be shared. There will be small meetings in the community and these will be announced. After these meetings, we will publish the results in order that other interested people may learn from our research.

Right to Refuse or Withdraw

You do not have to take part in this research if you do not wish to do so. You may also stop participating in the research at any time you choose. It is your choice and all of your rights will still be respected.

Who to Contact

If you have any questions you may ask them now or later, even after the study has started. If you wish to ask questions later, you may contact:

Job Wasonga
P.O. Box 6361 Kisumu.
Tel: 0722901434
Email: jwasonga@hotmail.com

This proposal has been reviewed and approved by KEMRI Scientific and Ethics Review Unit (SERU), which is a committee whose task it is to make sure that research participants are protected from harm. If you wish to find more about SERU, contact:

Scientific and Ethics Review Unit
Kenya Medical Research Institute (KEMRI)
P.O. Box 54840-00200
Nairobi.
Tel: 0722205901/0733400003
Email: info@kemri.org

You can ask me any more questions about any part of the research study, if you wish to. Do you have any questions?

PART II: Certificate of Consent

I have read the foregoing information, or it has been read to me. I have had the opportunity to ask questions about it and any questions that I have asked have been answered to my satisfaction. I consent voluntarily to participate as a participant in this research.

Print Name of Participant [illegible]

Signature of Participant ____________________

Date 18/07/2016
Day/month/year

If illiterate
A literate witness must sign (if possible, this person should be selected by the participant and should have no connection to the research team). Participants who are illiterate should include their thumb-print as well.

I have witnessed the accurate reading of the consent form to the potential participant, and the individual has had the opportunity to ask questions. I confirm that the individual has given consent freely.

Print name of witness [illegible] **AND** **Thumb print of participant**

Signature of witness ____________________

Date 18/07/2016
Day/month/year

Statement by the researcher/person taking consent
I have accurately read out the information sheet to the potential participant, and to the best of my ability made sure that the participant understands that the following will be done:

1.
2.
3.

I confirm that the participant was given an opportunity to ask questions about the study, and all the questions asked by the participant have been answered correctly and to the best of my ability. I confirm that the individual has not been coerced into giving consent, and the consent has been given freely and voluntarily.

A copy of this ICF has been provided to the participant.

Print Name of Researcher/person taking the consent [illegible]

Signature of Researcher /person taking the consent [illegible]

Date 18/07/2016
Day/month/year
